# Supplementary material for: Insight into genetic predisposition to chronic lymphocytic leukemia from integrative epigenomics
Source: Nat Commun. 2019 Aug 9;10:3615. doi: 10.1038/s41467-019-11582-2 (PMC6689100; doi:10.1038/s41467-019-11582-2)
Supplement: Supplementary file 2 — Description of Additional Supplementary Files [file 41467_2019_11582_MOESM2_ESM.pdf]

Description of Additional Supplementary Files

File Name: Supplementary Data 1
Description: **Position and characteristics of 42 risk loci.** The 42 risk loci analyzed in the study, including their sentinel SNP, the risk and non-risk allele, the position of the sentinel SNP, the linkage disequilibrium regions with an $r^2 \geq 0.2$ (LD0.2 region) and the topologically associating domains (TADs) covering the LD0.2 region, the probesets (HGU219 gene expression arrays) and genes within the TADs, and the number of patients per genotype for the different datasets.

File Name: Supplementary Data 2
Description: **Chromatin states in CLL at SNPs in LD0.2 with sentinel SNP.** Indicated are all SNPs in linkage disequilibrium ($r^2 \geq 0.2$) with the sentinel SNPs at the 42 risk loci. For each SNP the chromatin states in 7 CLL cases are indicated, as well as if they are located at active regulatory elements in one or more CLL cases.

File Name: Supplementary Data 3
Description: **Chromatin state enrichment in CLL cases for CLL, CRC and BC risk loci.** Indicated are the sentinel SNPs of the 42 CLL, 75 CRC and 165 BC risk loci and their linkage disequilibrium (LD) regions ($r^2 \geq 0.2$). Per CLL case and per SNP, the log2 fold change ofeach chromatin state in the LD region in comparison to the background chromatin distribution within the same CLL case, were calculated. Per SNP, the median and standard deviation of the log2 fold changes in 7 CLL cases are indicated.

File Name: Supplementary Data 4
Description: **H3K27ac signal in CLL in comparison to normal B cells.** For the 423 non-individual H3K27ac peaks in the linkage disequilibrium (LD) regions ($r^2 \geq 0.2$) of the 42 CLL risk loci the difference (log2 fold change, log2FC) in H3K27ac signal in CLL versus each of the normal B-cell subpopulations is indicated, including the standard error (lfcSE) and the 95% confidence interval (log2FC.CI) of the log2FC. H3K27ac signals in seven independent CLL cases were compared with H3K27ac signals in five normal B-cell subpopulations (three independent biological replicates each) using the Wald test in DESeq2 and were per comparison corrected for multiple testing. P-values, FDRs and the Wald statisticsare listed.

File Name: Supplementary Data 5
Description: **Overlap LD0.2 regions with previously defined *de novo* active regions in CLL.** The overlap between the linkage disequilibrium regions with an $r^2 \geq 0.2$ (LD0.2 region) of each sentinel SNP and the 498 *de novo* active regions in CLL as previously defined are shown, as well as the previously assigned target genes of these *de novo* active regions.

File Name: Supplementary Data 6
Description: **Characteristics and data availability of 502 CLLs.** Patient characteristics as well as the availability of the different datasets for all 502 CLL patients included in the study.

File Name: Supplementary Data 7
Description: **QTL analysis results.** Results of the QTL analysis for H3K27ac ChIP-seq data (a), ATAC-seq data (b), DNA methylation (c) and gene expression data (d), as well as the overlap among them.

File Name: Supplementary Data 8
Description: **Transcription factor binding analysis.** (a) For allvariants in linkage disequilibrium ($r^2 \geq 0.2$) with the sentinel SNPs at CLL risk loci that are located within the ATAC-seq peaks that were assessed for the accessibility QTLs analysis, the bound transcription factors (TFs) in lymphoblastoid cell lines are indicated as well as the motifs that show altered TF binding using MotifbreakR analysis. (b) Extended results of the MotifbreakR analysis. MotifbreakR analysis was only performed at bi-allelic variants.

File Name: Supplementary Data 9
Description: **SNPs in LD0.2 overlapping with ATAC-seq peaks and allelic imbalance results.** Indicated are all SNPs in linkage disequilibrium ($r^2 \geq 0.2$) with the sentinel SNPs at the 42 risk loci that overlap with ATAC-seq peaks and the results of the allelic imbalance analysis. For each SNP the position of the ATAC-seq peak is indicated as well as if this peak represents an accessibility QTL (including the p-value and FDR for the accessibility QTL analysis). For all SNPs that were analysed for allelic imbalance in chromatin accessibility, the number of heterozygous patients are indicated, as well as the number of reads containing the different nucleotides, the allele ratio for the reference allele (the effect size is the deviation of the observedratio from the expected ratio of 0.5), its confidence interval, the p-value and FDR for allelic imbalance.

File Name: Supplementary Data 10
Description: **Capture Hi-C interactions between LD-defined CLL risk loci and promoters of target genes.** Listed are the target genes that interact in CLL, naive and total B cellsat the three-dimensional chromatin level with regions within the LD-defined CLL risk loci. Only interactions within the topologically associating domains (TADs) are listed.

File Name: Supplementary Data 11
Description: **Potential functional variants.** List of risk loci, indicated by their sentinel SNP, with the genomic regionsharbouring candidate risk variants (accessibility QTLs and/or regions showing allelic imbalance for chromatin accessibility) and the potential functional variants located within these regions (in LD ($r^2 \geq 0.2$) with the sentinel SNP). In addition the altered TF binding motifs determined using MotifbreakR are indicated for each risk variant as well as if TFs are bound to these loci in lymphoblastoid cell lines (LCLs). Furthermore, the potential target genes per risk locus are shown, as determined using eQTL analysis. Genomic co-ordinates are listed according to GRCh38.

File Name: Supplementary Data 12
Description: **Background regions for chromatin state enrichment.** Listed are the genomic regions that served as the background for the chromatin state enrichment analysis.
