# Supplementary material for: Insight into genetic predisposition to chronic lymphocytic leukemia from integrative epigenomics
Source: Nat Commun. 2019 Aug 9;10:3615. doi: 10.1038/s41467-019-11582-2 (PMC6689100; doi:10.1038/s41467-019-11582-2)
Supplement: Supplementary file 15 — Reporting Summary [file 41467_2019_11582_MOESM15_ESM.pdf]

nature research

Corresponding author(s): José I. Martín-Subero and Richard Houlston

# Reporting Summary

Nature Research wishes to improve the reproducibility of the work that we publish. This form provides structure for consistency and transparency in reporting. For further information on Nature Research policies, see Authors & Referees and the Editorial Policy Checklist.

## Statistical parameters

When statistical analyses are reported, confirm that the following items are present in the relevant location (e.g. figure legend, table legend, main text, or Methods section).

| n/a | Confirmed | |
|---|---|---|
| ☐ | ☒ | The exact sample size (*n*) for each experimental group/condition, given as a discrete number and unit of measurement |
| ☐ | ☒ | An indication of whether measurements were taken from distinct samples or whether the same sample was measured repeatedly |
| ☐ | ☒ | The statistical test(s) used AND whether they are one- or two-sided<br>*Only common tests should be described solely by name; describe more complex techniques in the Methods section.* |
| ☐ | ☒ | A description of all covariates tested |
| ☐ | ☒ | A description of any assumptions or corrections, such as tests of normality and adjustment for multiple comparisons |
| ☐ | ☒ | A full description of the statistics including central tendency (e.g. means) or other basic estimates (e.g. regression coefficient) AND variation (e.g. standard deviation) or associated estimates of uncertainty (e.g. confidence intervals) |
| ☐ | ☒ | For null hypothesis testing, the test statistic (e.g. *F*, *t*, *r*) with confidence intervals, effect sizes, degrees of freedom and *P* value noted<br>*Give P values as exact values whenever suitable.* |
| ☒ | ☐ | For Bayesian analysis, information on the choice of priors and Markov chain Monte Carlo settings |
| ☒ | ☐ | For hierarchical and complex designs, identification of the appropriate level for tests and full reporting of outcomes |
| ☒ | ☐ | Estimates of effect sizes (e.g. Cohen's *d*, Pearson's *r*), indicating how they were calculated |
| ☒ | ☐ | Clearly defined error bars<br>*State explicitly what error bars represent (e.g. SD, SE, CI)* |

*Our web collection on statistics for biologists may be useful.*

## Software and code

Policy information about availability of computer code

| | |
|---|---|
| Data collection | Not applicable. |
| Data analysis | The following software tools were used in the manuscript: R, LDlink, DEseq2, IMPUTE2 v2.3, PLINK v1.9, SAMtools, Matrix eQTL, bowtie2 v2.2.6, HiCUP v0.5.9, CHiCAGO v1.1.8, WASP bwa0.7.7, motifbreakR package, SNPTESTv2.5, META v1.6. |

For manuscripts utilizing custom algorithms or software that are central to the research but not yet described in published literature, software must be made available to editors/reviewers upon request. We strongly encourage code deposition in a community repository (e.g. GitHub). See the Nature Research guidelines for submitting code & software for further information.

## Data

Policy information about availability of data

All manuscripts must include a data availability statement. This statement should provide the following information, where applicable:

- Accession codes, unique identifiers, or web links for publicly available datasets
- A list of figures that have associated raw data
- A description of any restrictions on data availability

All raw data for this study was mined from previous studies12-14,35 and has been deposited at the European Genome-Phenome Archive (EGA, http://

www.ebi.ac.uk/ega/), which is hosted at the European Bioinformatics Institute (EBI), under accession numbers EGAS00000000092, EGAD00001004046, EGAS00001000272 and EGAS00001001911.

# Field-specific reporting

Please select the best fit for your research. If you are not sure, read the appropriate sections before making your selection.

☒ Life sciences ☐ Behavioural & social sciences ☐ Ecological, evolutionary & environmental sciences

For a reference copy of the document with all sections, see nature.com/authors/policies/ReportingSummary-flat.pdf

# Life sciences study design

All studies must disclose on these points even when the disclosure is negative.

| | |
|---|---|
| Sample size | As far as we are aware, there is no established method to determine the optimal sample size for QTL analysis using data of primary samples as reported in our manuscript. To be on the safe side, we included a total of respectively 97, 99, 452 and 486 primary CLL samples for H3K27ac, ATAC-seq, RNA expression and DNA methylation QTL analysis, which is larger than previously published reports on QTL analysis in primary patient samples. |
| Data exclusions | Genotyping data from the 502 CLL cases were subject to standard quality control, resulting in the exclusion of 14 cases (see supplementary table 6) prior to imputation because of non-European ancestry (using the HapMap version 2 CEU, JPT/CHB and YRI populations as reference), excessive heterozygosity or relatedness. One CLL sample (CLL618) where the genotypes assigned by imputation and WGS showed <90% concordance for the sentinel SNPs was excluded from all analyses. |
| Replication | Experimental replication was not attempted. |
| Randomization | Due to the experimental design and goals of our resource article sample randomization was not neccessary. |
| Blinding | Due to the experimental design and goals of our resource article sample blinding was not neccessary. |

# Reporting for specific materials, systems and methods

## Materials & experimental systems

| n/a | Involved in the study |
|---|---|
| ☒ | ☐ Unique biological materials |
| ☒ | ☐ Antibodies |
| ☒ | ☐ Eukaryotic cell lines |
| ☒ | ☐ Palaeontology |
| ☒ | ☐ Animals and other organisms |
| ☐ | ☒ Human research participants |

## Methods

| n/a | Involved in the study |
|---|---|
| ☒ | ☐ ChIP-seq |
| ☒ | ☐ Flow cytometry |
| ☒ | ☐ MRI-based neuroimaging |

## Human research participants

Policy information about studies involving human research participants

| | |
|---|---|
| Population characteristics | The clinical and biological characteristics of the 502 MBL/SLL/CLL patients studied are detailed in Supplementary Table 6. Cases were defined as IGHV-mutated when the identity of immunoglobulin genes was <98%. All tumor samples were obtained pre-therapy. Age range: 18-93, median = 62. Gender: 293 males, 203 females, 6 unknown. IGHV-mutation status: 176 unmutated, 315 mutated, 1 biclonal and 10 unknown. Disease: 428 CLLs, 21 SLLs and 53 MBLs. |
| Recruitment | The patients within this study were recruited within the International Cancer Genome Consortium (Chronic Lymphocytic Leukemia Genome consortium). |
